# Expression of SGLT1 in Human Hearts and Impairment of Cardiac Glucose Uptake by Phlorizin during Ischemia-Reperfusion Injury in Mice

**DOI:** 10.1371/journal.pone.0130605

**Published:** 2015-06-29

**Authors:** Yusuke Kashiwagi, Tomohisa Nagoshi, Takuya Yoshino, Toshikazu D. Tanaka, Keiichi Ito, Tohru Harada, Hiroyuki Takahashi, Masahiro Ikegami, Ryuko Anzawa, Michihiro Yoshimura

**Affiliations:** 1 Division of Cardiology, Department of Internal Medicine, The Jikei University School of Medicine, Tokyo, Japan; 2 Department of Pathology, The Jikei University School of Medicine, Tokyo, Japan; Rutgers New Jersey Medical School, UNITED STATES

## Abstract

**Objective:**

Sodium-glucose cotransporter 1 (SGLT1) is thought to be expressed in the heart as the dominant isoform of cardiac SGLT, although more information is required to delineate the subtypes of SGLTs in human hearts. Moreover, the functional role of SGLTs in the heart remains to be fully elucidated. We herein investigated whether SGLT1 is expressed in human hearts and whether SGLTs significantly contribute to cardiac energy metabolism during ischemia-reperfusion injury (IRI) via enhanced glucose utilization in mice.

**Methods and Results:**

We determined that SGLT1 was highly expressed in both human autopsied hearts and murine perfused hearts, as assessed by immunostaining and immunoblotting with membrane fractionation. To test the functional significance of the substantial expression of SGLTs in the heart, we studied the effects of a non-selective SGLT inhibitor, phlorizin, on the baseline cardiac function and its response to ischemia-reperfusion using the murine Langendorff model. Although phlorizin perfusion did not affect baseline cardiac function, its administration during IRI significantly impaired the recovery in left ventricular contractions and rate pressure product, associated with an increased infarct size, as demonstrated by triphenyltetrazolium chloride staining and creatine phosphokinase activity released into the perfusate. The onset of ischemic contracture, which indicates the initiation of ATP depletion in myocardium, was earlier with phlorizin. Consistent with this finding, there was a significant decrease in the tissue ATP content associated with reductions in glucose uptake, as well as lactate output (indicating glycolytic flux), during ischemia-reperfusion in the phlorizin-perfused hearts.

**Conclusions:**

Cardiac SGLTs, possibly SGLT1 in particular, appear to provide an important protective mechanism against IRI by replenishing ATP stores in ischemic cardiac tissues via enhancing availability of glucose. The present findings provide new insight into the significant role of SGLTs in optimizing cardiac energy metabolism, at least during the acute phase of IRI.

## Introduction

The derangement of cardiac energy substrate metabolism plays a key role in the pathogenesis of heart disease [1]. Although the utilization of fatty acids is the predominant metabolic pathway in the normal adult heart, glucose becomes an important preferential substrate for metabolism and ATP generation under specific pathological conditions, such as ischemia, as it provides greater efficiency for producing high energy products per oxygen molecule consumed compared to fatty acids. During ischemia, impaired oxidative phosphorylation allows glycolysis to become a major mechanism by which the heart maintains the ATP concentration. Therefore, the acceleration of glycolysis and glucose utilization in the ischemic myocardium may be cardioprotective, with an improved cardiac functional recovery after ischemia-reperfusion injury (IRI) [1–5].

Glucose utilization is initiated primarily via glucose transporters, and glucose transport appears to be the rate-limiting step in glycolytic flux in the heart [6]. Glucose transporters are divided into two major families: facilitated glucose transporters (GLUTs) and sodium-coupled active transporters (SGLTs). The regulation of the GLUT expression and the functional roles of these transporters in the heart have been intensively investigated in a variety of *in vitro* and *in vivo* models [2,4,5,7,8]. However, less is known about the role and functional significance of SGLTs in the heart. Among SGLTs, SGLT1 is thought to be expressed as the dominant isoform of cardiac SGLT [9–12], although more information is necessary to delineate the subtypes of SGLTs in human hearts. In this study, we first investigated whether SGLT1 is expressed in hearts, including human hearts. Second, we studied whether SGLTs significantly contribute to glucose uptake in the heart as the initial rate-limiting step for cardiac energy metabolism during IRI using the *ex vivo* murine Langendorff model with perfusion with the non-selective SGLT inhibitor, phlorizin.

## Materials and Methods

### Experiments in Langendorff hearts

All animal procedures conformed to the National Institutes of Health Guide for the Care and Use of Laboratory Animals and were approved by the Animal Research Committee at the Jikei University School of Medicine (21-009C3). Eight to ten-week old male ICR mice (weight: 36 to 38 g, fasted for 6–8 hours) were heparinized (1000 IU/kg, i.p.) and anesthetized (pentobarbital, 60 mg/kg, i.p.) in order to eliminate suffering. The heart was then rapidly excised, and the aorta was cannulated onto a Langendorff apparatus, followed by retrograde-perfusion at a constant pressure (80 mmHg) with modified Krebs-Henseleit buffer, as previously described [3,13–15]. A water-filled balloon catheter was introduced into the left ventricle to record various hemodynamic parameters.

### Ischemia-reperfusion model

**Fig 1:**
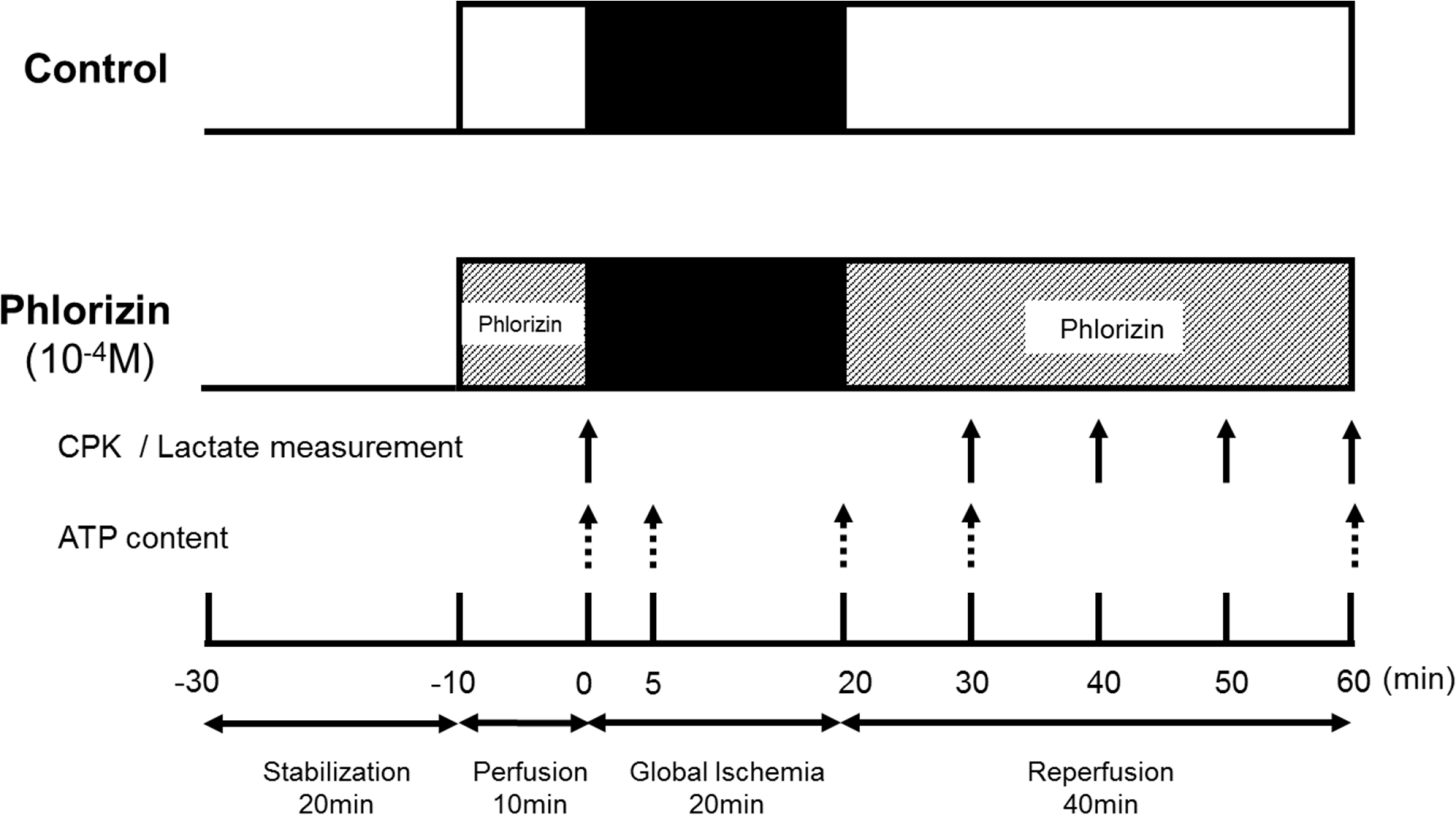
Experimental protocol showing the duration and time course of ischemia/reperfusion. CPK activity and lactate output released into the perfusate were measured at the point in the protocol indicated by the solid arrows. The individual perfused hearts were homogenized for measurement of the tissue ATP content at the point in the protocol indicated by the dotted arrows. min, minutes.

The experimental protocols are shown in Fig 1. After a stabilization period of 20 minutes, the control hearts were perfused for another 10 minutes before ischemia-reperfusion in order to measure the baseline pre-ischemia cardiac function. Subsequently, global ischemia was applied by eliminating flow for 20 minutes followed by 40 minutes of reperfusion. In the phlorizin-perfused group, 10^−4^ mol/L of phlorizin (Sigma-Aldrich, Tokyo, Japan) was added to the buffer during the 10-minute pre-ischemia perfusion and 40-minute reperfusion periods. Phlorizin was dissolved in dimethylsulfoxide (DMSO), and the solvent concentration was identically maintained in the control group. For the immunoblotting analysis, the individual perfused hearts after ischemia-reperfusion or the hearts simply perfused for an equivalent interval of the ischemia-reperfusion protocol (namely, 90 minutes) were snap frozen in liquid nitrogen and stored at -80°C prior to protein extraction. Triphenyltetrazolium chloride (TTC) staining was performed to determine the myocardial infarct size using the individual perfused hearts after ischemia-reperfusion, as previously described [3].

### Glucose uptake in Langendorff hearts

After a stabilization period, the hearts were perfused for another 20 minutes with phosphate free Krebs-Henseleit buffer (in mM:118 NaCl, 25 NaHCO3, 5.3 KCl, 2.5 CaCl2, 1.2 MgSO4, 0.5 EDTA, 5 pyruvate and 5 glucose). And then, the perfusate was switched to a buffer containing 3 mM of the glucose analog 2-deoxy-D-glucose (2-DG), instead of glucose, and 1.2 mM KH_2_PO_4_ to replenish the phosphate pool for 30min. The rate of glucose transport in Langendorff perfused hearts was measured by detection of the amount of 2-deoxyglucose (2-DG). For measurement of 2-DG content, 10mg of Langendorff perfused heart samples was homogenized in 500 μ of 10mM Tris-HCL (pH 8.0), heated (95°C, 15 minutes), and then centrifuged (17800g, 15 minutes, 4°C). The resulting supernatants were diluted (20×) with 10 mM Tris-HCl (pH 8.0) followed by detection of the amount of 2-DG using a 2-DG Uptake Measurement kit (Cosmo Bio Co. Ltd., Tokyo, Japan) according to manufacturer’s protocol as previously described [16,17].

### Cardiac enzyme measurement and biochemical assay

Creatine phosphokinase (CPK) levels were measured in the effluent at the time points indicated in the experimental protocols (Fig 1) using an enzymatic activity assay, as previously described [15]. Values were corrected for coronary flow and heart weight [CPK (U/l) x coronary flow (l/min) / heart weight (g) = U/min/g] [3]. Lactate output was determined by measuring the perfusate lactic acid concentration at the time points indicated in the experimental protocols (Fig 1) using enzymatic method [18]. Values were corrected for coronary flow and heart weight [Lactic acid (μmol/l) x coronary flow (l/min) / heart weight (g) = µmol /min/g].

### Cardiac muscle fractionation

The preparation and fractionation of total membranes from cardiac muscles was performed using ProteoExtract Transmembrane Protein Extraction Kit (Novagen, Darmstadt, Germany) according to the manufacturer’s protocol. In brief, 25–50 mg of frozen perfused heart tissue was homogenized in ice-cold Extraction Buffer 1 supplemented with Protease Inhibitor Cocktail Set III. The fragmented tissue was subsequently incubated for 10 minutes at 4°C with gentle agitation and then centrifuged (1000 g, 5 minutes). The supernatants (cytosolic fraction) were removed, and the pellets were resuspended in 5 ml ice-cold Phosphate Buffered Saline (PBS) and centrifuged (1000 g, 5 minutes). After carefully removing the supernatants, the pellets were resuspended in 0.2 ml of ice-cold Extraction Buffer 2B supplemented with Protease Inhibitor Cocktail Set III. Following 15 minutes incubation at 4°C with gentle agitation, the materials were centrifuged (16000 g, 15 minutes), and the supernatants, enriched in integral membrane proteins, were transferred to a fresh tube.

The plasma membrane fraction was isolated using Minute Plasma Membrane Protein Isolation Kit (Invent Biotechnologies, Eden Prairie, MN) according to the manufacture’s protocol as previously described [19]. All steps were performed at 4°C. In brief, 30mg of frozen heart tissue were lysed in buffer A and placed in a filter cartridge. After centrifugation (14,000 rpm, 30 seconds), the pellets were resuspended in buffer A and centrifuged (3,000 rpm, 1 minute). The supernatant was then centrifuged (14,000 rpm, 10 minutes). After the supernatant (cytosol protein fraction) was removed, the pellets (total membrane fraction) were resuspended in buffer B and centrifuged (10,000 rpm, 5 minutes). The resultant pellets were collected separately as organelle membrane protein. The supernatant was centrifuged (15,000 rpm, 15 minutes), and the pellets were collected in 0.5% Triton X-100/PBS as plasma membrane protein fraction.

After either total or plasma membrane fractionation, the protein concentrations were determined according to a Bradford assay, and equal amounts of protein were loaded for immunoblotting.

### Immunoblotting

Immunoblotting was performed as previously described [15,20,21] with rabbit polyclonal anti-SGLT1 (for mouse tissue: 1:200, Santa Cruz Biotechnology #sc98974, CA, USA; for human tissue: 1:1000, Medical and Biological Laboratories Co. #BMP022, Nagoya, Japan), or mouse monoclonal anti-α1 Na-K-ATPase (1:1000, Abcam #ab7671, Cambridge, MA, USA). The signals were detected using chemiluminescence.

### Tissue ATP content measurement

The ATP content in the heart tissue was determined using a firefly bioluminescence assay kit (AMERIC-ATP kit; Wako Pure Chemical Industries, Osaka, Japan) according to the manufacturer’s protocol. The assay included luciferase, which generates a stable luminescent signal proportional to the amount of ATP present [22]. Briefly, the individual perfused hearts were homogenized with 3.0 mL ice-cold Tris-EDTA (10 mmol/L Tris-HCl, pH 8.0 and 1 mmol/L EDTA)-saturated phenol. One ml of homogenate was transferred into 1.5-ml microtubes containing 200 μl chloroform and 150 μl de-ionized water. After being thoroughly shaken, the homogenate was centrifuged (10000 g, 5 minutes) at 4°C in order to achieve phase separation, and 50 μl of the upper aqueous phase was stored at -80°C. Measurement of the ATP levels was outsourced to Applied Medical Enzyme Research Institute Corporation (Tokushima, Japan).

### Glycogen content measurement

Glycogen content in the heart tissue was determined using Glycogen Colorimetric Assay kit (BioVision, Milpitas, CA) according to the manufacturer’s protocol. Langendorff-perfused heart tissues (10 mg) were homogenized with 200µl dH_2_O (on ice), boiled (100°C, 10 minutes), and centrifuged (18000g, 10 minutes). The supernatants (50 μl) were transferred to a 96-well plate. To hydrolyze the glycogen to glucose, 2μl of Hydrolysis Enzyme Mix were added and incubated (room temperature, 30 minutes). The samples were incubated with 50μl of Reaction Mix (Development buffer 46 µl, Development Enzyme Mix 2μl, OxiRed Probe 2μl) (room temperature, 30minutes), and then, absorbance was measured (optical density at 570 nm).

### Human tissues

Written informed consent was obtained from the next of kin for the use of postmortem tissue specimens in research, and all clinical investigations were conducted in accordance with the principles expressed in the Declaration of Helsinki. The use of human tissues was approved by the Ethics Committee of the Jikei University School of Medicine (25-122-7257). The samples were obtained from adult males and females between 47 and 75 years of age who underwent autopsies (Table 1). At autopsy, human heart tissues were cut from the ventricle and atrium, snap frozen in liquid nitrogen and stored at -80°C prior to protein extraction.

**Table 1:** Characteristic of human tissue donors.

Sex	Age (years)	The cause of death	Comorbidities	Postmortem time (hours)
M	47	Primary sclerosing cholangitis (Post-transplant liver failure)	Hemophagocytic syndrome, Ulcerative colitis	4.5
F	51	Myelodysplastic syndromes overt leukemia	Graft versus host disease, Chronic Active Epstein-Barr Virus infection	19
F	75	Acute myelogenous leukemia	Femoral neck fracture	10.5
M	63	Old myocardial infarction	Aortic valve stenosis, Post coronary artery bypass grafting, Aortic valve replacement, Percutaneous coronary intervention, Chronic kidney disease, Asbestos lung	12
M	50	Chronic myocarditis	Atrial flutter (Post ablation) Ventricular tachycardia, Post cardiac resynchronization therapy with defibrillator implant, Old cerebral infarction	8

### Immunohistochemistry

The frozen tissues were cut into small pieces (approximately 1mm^3^), laid in a cryomold (Sakura Finetek Japan Co., Ltd., Tokyo, Japan) filled with optimum cutting temperature compound (Muto Pure Chemicals Co., Ltd., Tokyo, Japan) and placed back into the deep freezer. Frozen sections (4 μm) were cut using a cryostat (CM1950; Leica, Wetzlar, Germany) and either immunostained or stained with hematoxylin and eosin. Immunohistochemistry was performed as previously described [23] with rabbit polyclonal anti-SGLT1(1:100, Abcam #ab14685, Cambridge, MA, USA) [24].

### Statistical analysis

The data are presented as the mean ± SEM of at least three independent experiments. The hemodynamic parameters and ATP content levels were compared using Student’s t-test. The CPK levels and %MI determined according to TTC staining were compared using Wilcoxon rank-sum test. A value of P<0.05 was considered to be significant.

## Results

### SGLT1 is highly expressed in human and murine hearts

**Fig 2:**
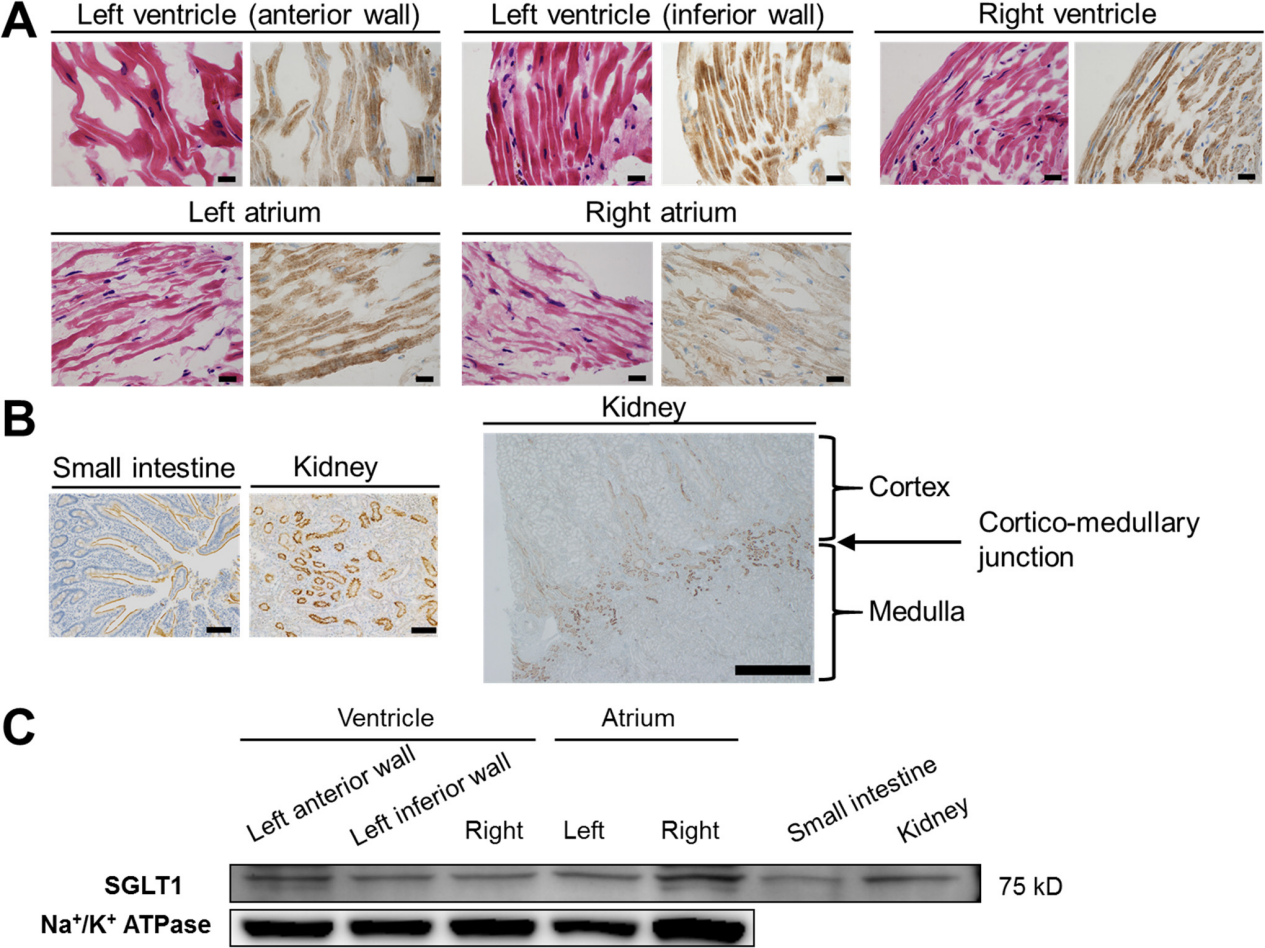
SGLT1 is highly expressed in human hearts. (A) Results of the immunohistochemical analysis of the SGLT1 expression in the various parts of the myocardium (in each panel: left, HE staining; right, immunostaining with an SGLT1 antibody) obtained from the human autopsied hearts (x40). Representative data from five independent patients are shown. Bars: 20 μm. (B) Immunohistochemical analysis of the SGLT1 expression using the same antibody in the brush border membrane of the human small intestine (left panel, x10, bar: 100 μm) and proximal tubule straight segment in the deep cortex and medullary rays (Cortico-medullary junction) of human kidneys (mid panel, x10, bar: 100 μm; right panel, x2, bar: 1mm) obtained from intraoperative samples shown as positive controls. (C) Representative immunoblots of SGLT1 in the membrane fraction from the indicated regions in the human autopsied hearts from four independent patients are shown. Total lysates extracted from the human autopsied small intestine and kidneys were immunoblotted as positive controls. Immunoblots of Na^+^/K^+^ ATPase from the same membrane are shown as a loading control for the membrane fraction.

**Fig 3:**
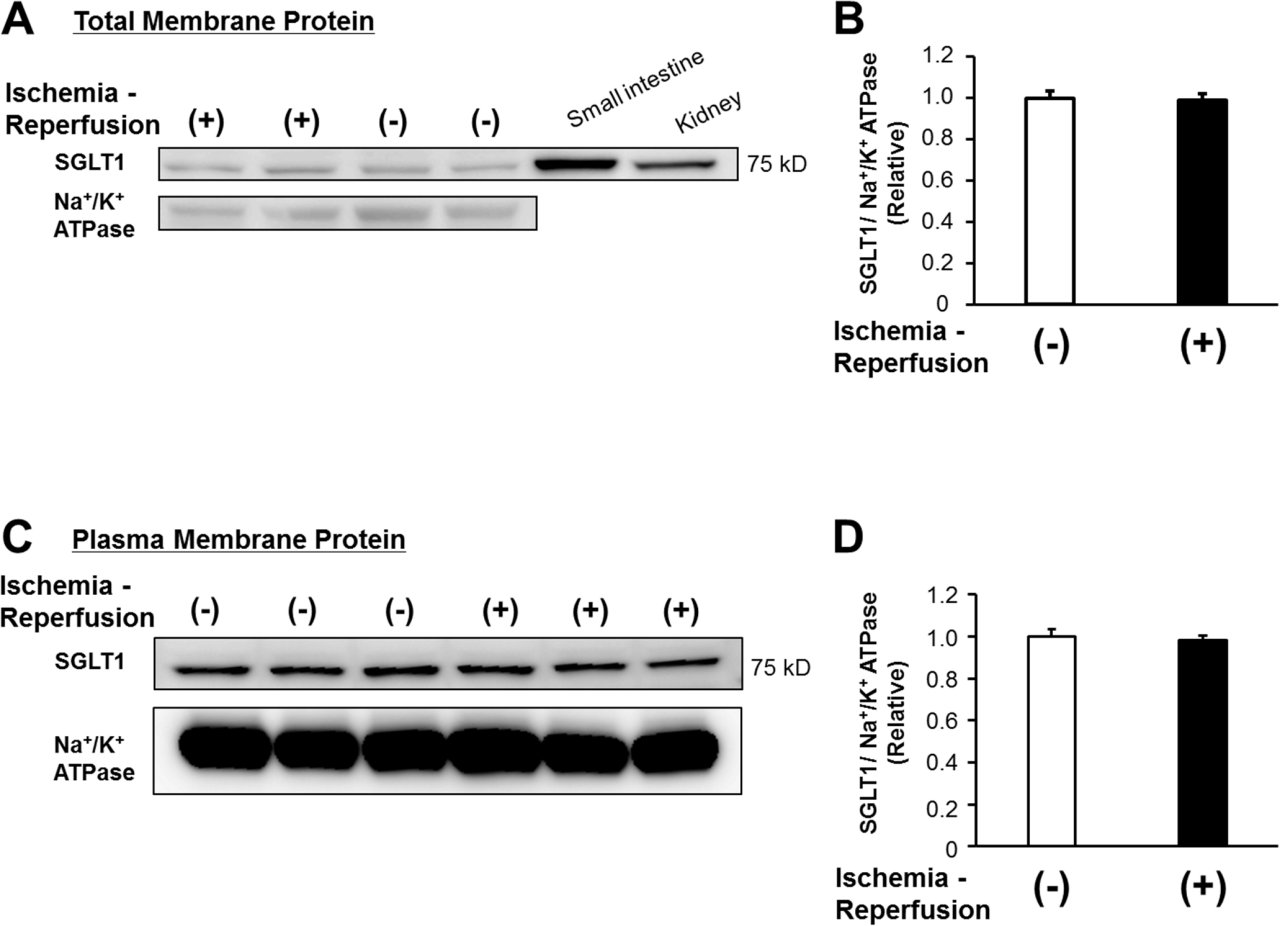
SGLT1 is expressed in the murine perfused hearts. (A) Representative immunoblots of SGLT1 in the total membrane fraction from the murine perfused hearts treated with or without ischemia-reperfusion are shown. Total lysates extracted from the murine small intestine and kidney were immunoblotted as positive controls. (B) Densitometric quantitation normalized to the level of control perfused hearts without ischemia-reperfusion is shown (n = 3 each). (C) Immunoblots of SGLT1 in the plasma membrane fraction from the murine perfused hearts with or without ischemia-reperfusion are shown. (D) Densitometric quantitation normalized to the level of control perfused hearts without ischemia-reperfusion is shown (n = 3 each). In both (A) and (C), immunoblots of Na^+^/K^+^ ATPase from the same membrane are shown as a loading control for the membrane fraction.

Previous reports indicated that SGLT1 is primarily expressed in the heart as well as the brush border membrane of the small intestine and proximal tubule straight segment in the kidneys [9,11,12,25–27]. In the heart, SGLT1 is thought to be localized to the sarcolemma in cardiomyocytes [9,10,28–32] and in capillaries [25,27]. In agreement with these findings, we determined on the immunohistochemical analyses that SGLT1 was substantially expressed in the whole heart tissues obtained from the human autopsied hearts (Fig 2A). Using the same SGLT1 antibody (which has previously been used for detection of SGLT1 in human tissue immunohistochemistry study [24]), SGLT1 was stained exclusively in the brush border membrane of the human small intestine as well as the proximal tubule straight segment in the deep cortex and medullary rays (cortico-medullary junction) of the human kidneys, but not in the cortex zone where SGLT2 is substantially expressed (Fig 2B), indicating the specificity of the SGLT1 antibody used in the current study. These findings are supported by the results of immunoblotting with membrane fractionation, which showed that SGLT1 was ubiquitously expressed in the human heart tissues obtained from the autopsy samples (Fig 2C). Similarly, SGLT1 was also clearly detected in both total and plasma membrane fraction of the murine Langendorff perfused hearts (Fig 3A and 3C, respectively). Ischemia-reperfusion did not significantly affect the transmembrane protein expression levels of SGLT1 (Fig 3B and 3D). These data demonstrate that SGLT1 is highly expressed in both human and murine hearts.

### Effects of phlorizin on cardiac functional recovery after IRI

**Fig 4:**
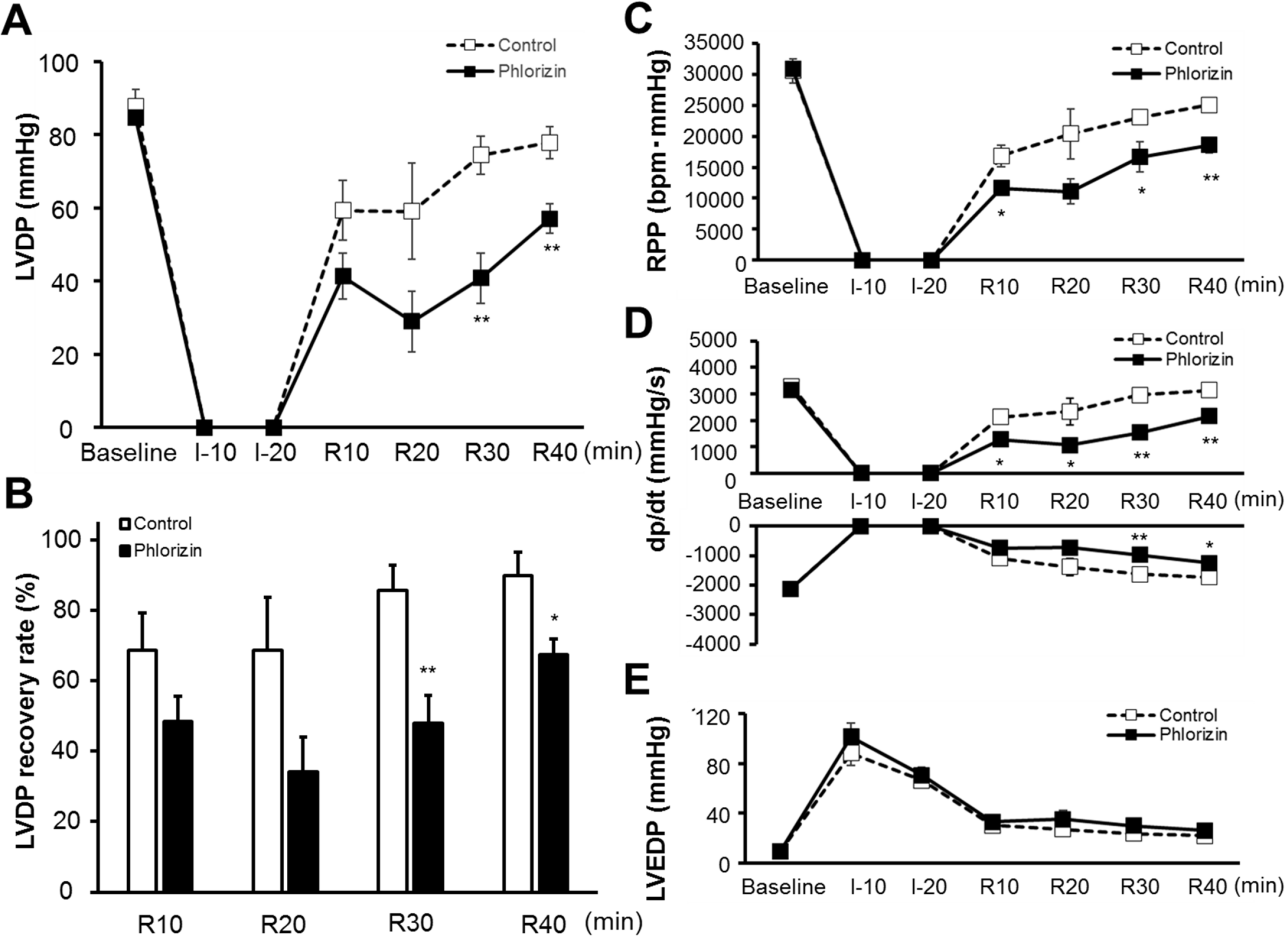
Cardiac functional recovery after ischemia-reperfusion injury. LVDP profiles during ischemia-reperfusion (A), LVDP recovery (percent of baseline) measured at the indicated time points (B), RPP profiles (C), positive and negative dp/dt profiles (D) and LVEDP profiles (E) during ischemia-reperfusion in the phlorizin-perfused hearts (filled square; n = 5) and control hearts (open square; n = 5). *P<0.05 and **P<0.01 versus the control group at each time point.

The baseline cardiac function measured at the end of the 10-minute pre-ischemia perfusion period was not significantly affected by the SGLTs inhibitor, phlorizin (Table 2). After 20-minute global ischemia followed by 40-minute reperfusion, the administration of phlorizin during IRI significantly reduced the left ventricular developed pressure (LVDP) recovery compared with that observed in the non-treated control hearts (67.3±4.5 versus 89.7±6.8% recovery from baseline, P<0.05, Fig 4A and 4B). Moreover, in order to consider the impact of the heart rate on the cardiac function, we measured the rate pressure product (RPP, calculated as LVDP × heart rate), which showed a significant reduction in the phlorizin-perfused hearts after IRI (18600±1290 versus 25100±1010 mmHg•bpm at the end of IRI, P<0.01, Fig 4C). In addition, the maximum rate of contraction (+dp/dt_max_) and maximum rate of relaxation (-dp/dt_min_) were impaired in the phlorizin-perfused hearts after IRI (the end of IRI, 2148±94 versus 3138±202 mmHg/s (P<0.01), and -1254±94 versus -1728±167 mmHg/s (P<0.05), respectively, Fig 4D). There were no significant differences in the left ventricular end-diastolic pressure (LVEDP) during IRI between two groups (Fig 4E).

**Table 2:** Baseline cardiac function of *ex vivo* perfused hearts.

	Control (n = 8)	Phlorizin (n = 9)
LVSP, mmHg	96.1±2.9	92.7±0.9
LVEDP, mmHg	8.9±0.4	9.4±0.3
LVDP, mmHg	87.3±2.8	83.3±1.0
+dp/dt, mmHg/s	3131±155	2921±136
-dp/dt, mmHg/s	-2216±82	-2035±63
HR, bpm	359±15	350±10
RPP, mmHg・bpm	31200±1310	29200±939
Coronary flow, ml/min	2.92±0.49	3.91±0.42

LVSP, left ventricular systolic pressure; LVEDP, left ventricular end-diastolic pressure; LVDP, left ventricular developed pressure; HR, heart rate; RPP, rate pressure product.

### Cardiac injury after ischemia-reperfusion

**Fig 5:**
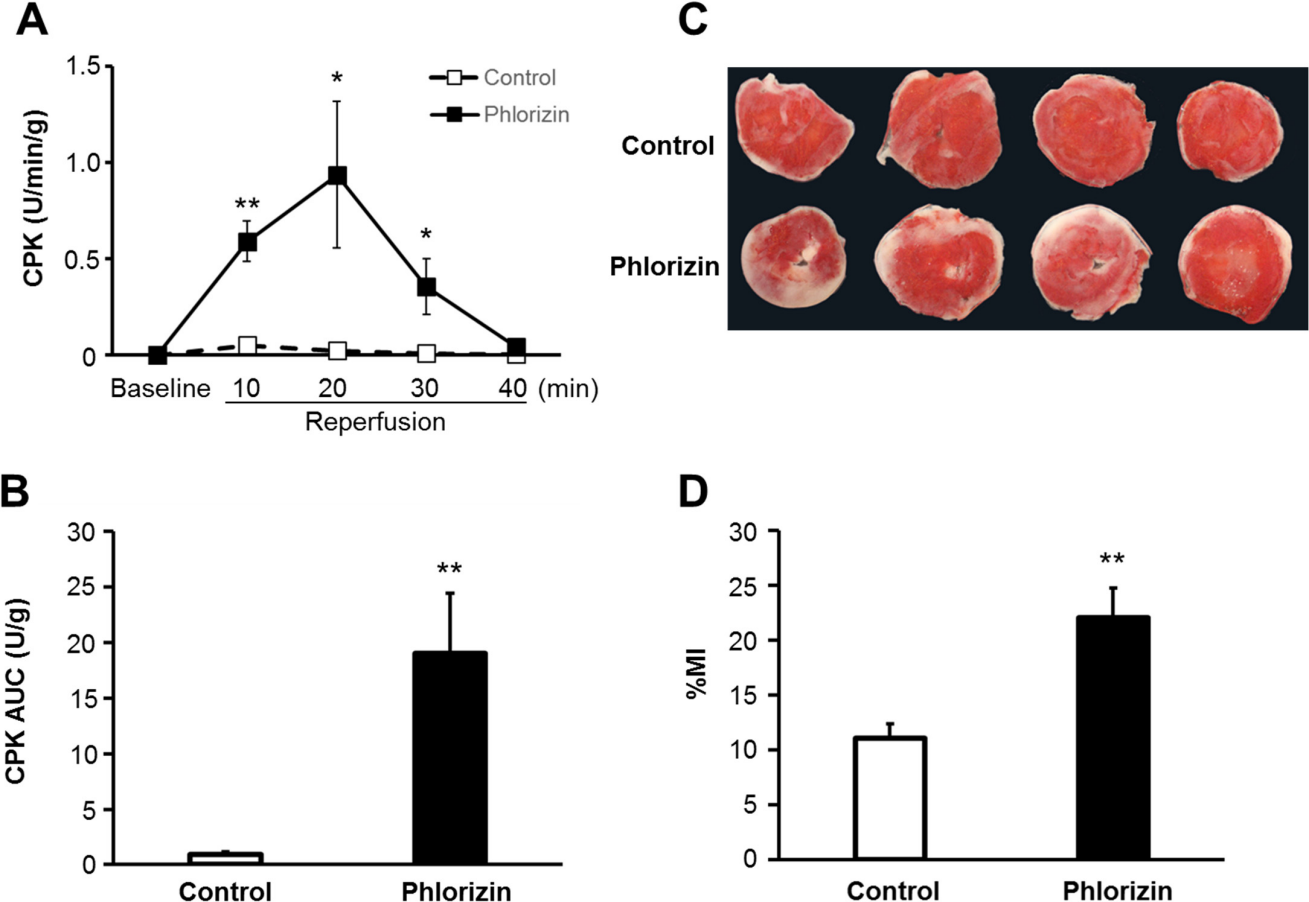
Phlorizin increased myocardial injury after ischemia-reperfusion. (A) CPK profiles in the effluent collected during the reperfusion period. (B) Area under the curve (AUC) was calculated from the CPK profile shown in (A). (phlorizin-perfused hearts, n = 11; control hearts, n = 10). (C) Micrograph showing representative TTC staining of cardiac sections obtained from the control (top row) and phlorizin-perfused hearts (bottom row). (D) Effects on quantitated cumulative infarct area size in the phlorizin-perfused hearts (n = 9) compared with that observed in the control group (n = 8). %MI, myocardial infarct area/ventricular area. *P<0.05 and **P<0.01 versus control.

The activity of CPK released into the perfusate during reperfusion was measured as an index of myocardial injury. During the baseline pre-ischemia perfusion period, no CPK activity was detectable. After IRI, the CPK activity was significantly increased in the phlorizin-perfused hearts compared with that observed in the control hearts at 10, 20 and 30 minutes of reperfusion (0.59±0.11 versus 0.05±0.02 U/min/g, P<0.01; 0.94±0.38 versus 0.02±0.01 U/min/g, P<0.05; 0.35±0.14 versus 0.01±0.004 U/min/g, P<0.05, respectively) (Fig 5A). Moreover, the total amount of CPK released during the entire reperfusion period, as indicated by the CPK area under the curve (AUC) for the CPK level, was significantly increased by phlorizin perfusion (19.0±5.4 versus 0.9±0.3 U/g, P<0.01, Fig 5B). This finding correlated with larger infarcts in the phlorizin-perfused hearts compared with those noted in the control hearts (22.1±2.7 versus 11.1±1.3%, P<0.01, Fig 5C and 5D).

### Effects of phlorizin on myocardial glucose utilization during IRI

**Fig 6:**
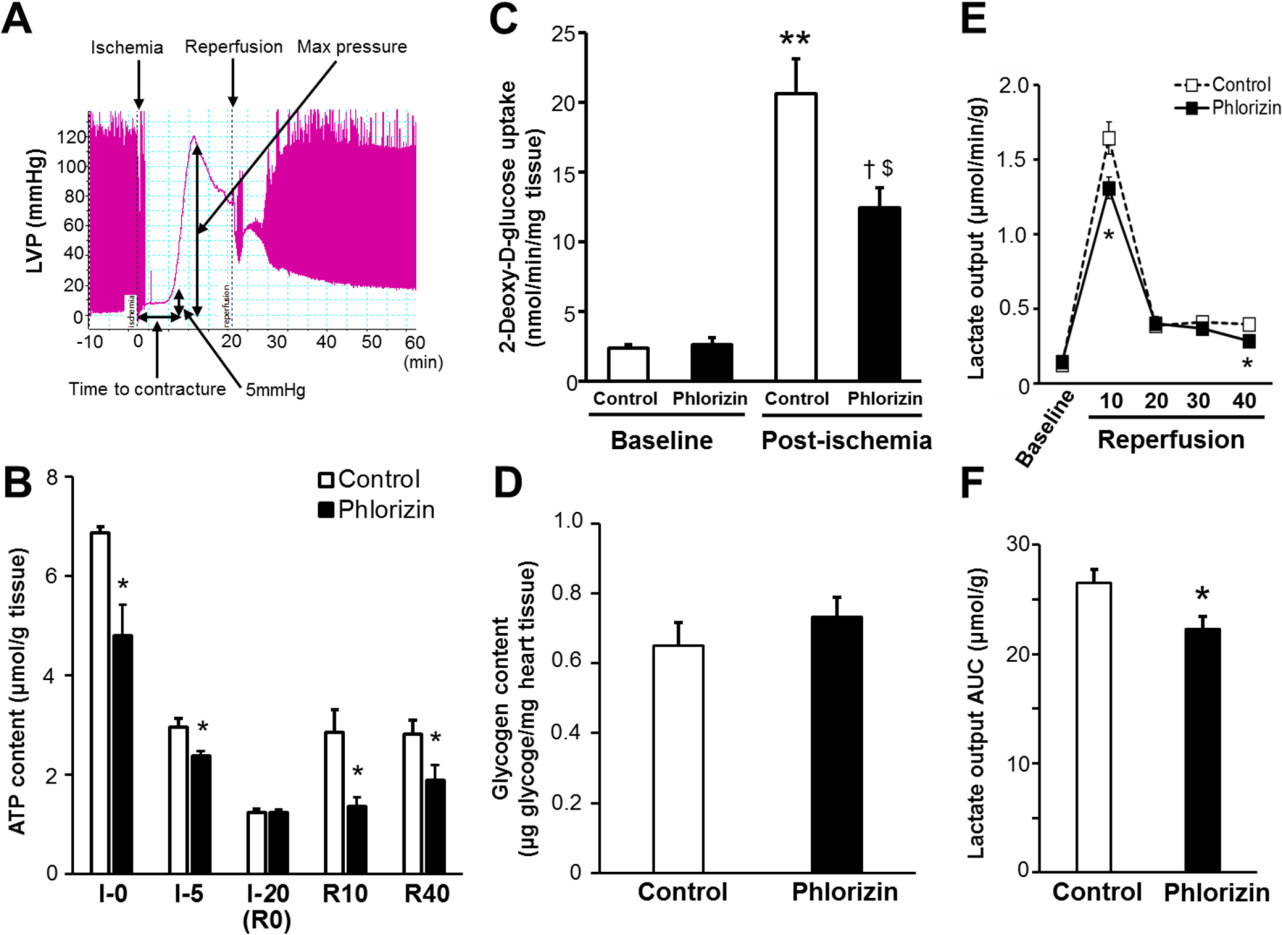
Phlorizin reduced tissue ATP content in the heart, associated with decreased glucose uptake and glycolytic flux during IRI. (A) Ischemic contracture was observed as a sigmoid increase in end-diastolic pressure, the onset and extent of which was recorded. The definitions of the individual parameters are shown. (B) The tissue ATP content in the hearts measured at the indicated time points (Ischemia 0 minutes: n = 4 each, Ischemia 5 minutes: n = 4 each, Ischemia 20 minutes: phlorizin-perfused; n = 4, control; n = 7, Reperfusion 10 minutes: phlorizin-perfused; n = 7, control; n = 8, Reperfusion 40 minutes: phlorizin-perfused; n = 7, control; n = 8). *P<0.05 versus control. (C) Glucose uptake in the hearts perfused with or without phlorizin under the pre-ischemic baseline condition (n = 6 each) and post-ischemic condition measured at 10-minute reperfusion following 20-minute global ischemia (phlorizin-perfused; n = 7, control; n = 6). **P<0.01 versus the control hearts at baseline; ^†^P<0.01 versus the phlorizin-perfused hearts at baseline; ^$^P<0.05 versus the control hearts at post-ischemia. (D) Glycogen content in the perfused hearts under the baseline condition prior to global ischemia (n = 4 each). (E) Lactate output profiles in the effluent collected during the reperfusion period (n = 10 each). *P<0.05 versus control. (F) AUC was calculated from the lactate output profiles shown in (E) (n = 10 each). *P<0.05 versus control.

Ischemic contracture, recorded as an increase in diastolic pressure above baseline followed by a continuous rise in pressure after global ischemia, is thought to be initiated by a decrease in the cardiac tissue ATP content [33–35]. The onset of contracture was defined as a 5-mmHg sigmoid increase in the end-diastolic pressure [35], and the time from the start of global ischemia to the onset of contracture was assessed (Fig 6A). In the present study protocol (Fig 1), all of the hearts with or without phlorizin perfusion went into ischemic contracture during the 20-minute global ischemia period. Although the maximum contracture pressure and time to maximum pressure values (as indicated in Fig 6A) were not significantly different, ischemic contracture appeared significantly earlier in the phlorizin-perfused hearts than in the control hearts (Table 3), suggesting that phlorizin administration during IRI promotes ATP depletion in the heart. Consistent with these findings, the tissue ATP content measured at 0 minute (right before stopping perfusion) and 5 minutes of global ischemia was significantly reduced in the phlorizin-perfused hearts compared with that measured in the control hearts (0 minutes:4.81±0.61 versus 6.87±0.13 μmol/g tissue, P<0.05; 5 minutes: 2.38±0.10 versus 2.96±0.18 μmol/g tissue, P<0.05, Fig 6B), although a comparably severe reduction was observed at the end of ischemia in both groups (1.24±0.06 versus 1.24±0.07 μmol/g tissue, Fig 6B). In addition, the tissue ATP content was also significantly reduced in the phlorizin-perfused hearts during both the early and late phases of reperfusion (reperfusion 10 minutes:1.37±0.17 versus 2.86±0.45 μmol/g tissue, P<0.05; reperfusion 40 minutes: 1.90±0.30 versus 2.83±0.27 μmol/g tissue, P<0.05, Fig 6B), indicating that there was an impairment in the replenishment of the ATP stores in the phlorizin-perfused hearts during IRI. The reduction in aerobic ATP formation accelerates glucose uptake as well as glycolysis, leading to an increase in contribution of glycolysis as a source of ATP production [1,5,36]. To investigate the mechanism by which the heart maintains the ATP concentrations during IRI, we measured glucose uptake, one of the major determinants of myocardial glucose utilization, in an *ex vivo* Langendorff perfusion system using a glucose analog, 2-deoxy-D-glucose (2-DG) (Fig 6C). At baseline, the rate of glucose uptake was similar between the phlorizin-perfused hearts and the control hearts (2.65±0.52 versus 2.42±0.20 nmol/min/mg heart tissue). Moreover, the tissue content of glycogen (endogenous glucose storage used to sustain ATP synthesis through glycolysis) was also similar between the phlorizin-perfused and control hearts at baseline (0.73±0.06 versus 0.65±0.07μg/mg heart tissue, Fig 6D). In contrast, during ischemia-reperfusion, the cardiac glucose uptake was dramatically enhanced by the ischemic insult, which was significantly attenuated in the phlorizin-perfused hearts (20.6±2.53 versus 12.5±1.43 nmol/min/mg heart tissue, P<0.05, Fig 6C). To evaluate myocardial glycolytic flux during IRI in an *ex vivo* model, total lactate production during ischemia-reperfusion were measured (Fig 6E and 6F) [5,18,22,34]. The lactate output during reperfusion following global ischemia was significantly decreased in the phlorizin-perfused hearts compared with that measured in the control hearts (Total lactate output (AUC): 22.3±1.1 versus 26.5±1.3 μmol/g tissue, P<0.05, Fig 6F). These data suggest that the inhibition of SGLTs during IRI leads to a reduction in glucose uptake, as well as glycolytic flux in the heart (namely, myocardial glucose utilization), thus resulting in impaired cardiac energy metabolism, which is manifested by a decrease in myocardial ATP replenishment.

**Table 3:** Ischemic contracture profiles during global-ischemia.

	Control (n = 8)	Phlorizin (n = 9)
Time to onset of contracture, sec	364±27	288±21 *
Maximum pressure, mmHg	102±6	100±7
Time to maximum pressure, sec	599±47	528±35

*P < 0.05 versus control.

## Discussion

In the present study, we found that SGLT1 is highly expressed in the heart and that the inhibition of SGLTs results in an impaired cardiac functional recovery and increased myocardial injury after ischemia-reperfusion. These findings were associated with significant reductions in the glucose uptake, which was in accordance with a decrease in the tissue ATP content in the heart during ischemia-reperfusion. This suggests that SGLTs play an important role in cardioprotection against IRI by maintaining the tissue ATP concentration, possibly as a result of enhanced glucose utilization in the heart.

The enhanced glucose uptake and glycolysis under pathological conditions, such as IRI, have been shown to be cardioprotective, thought to be largely mediated via the expression of GLUT1 as a basal transporter [7] and GLUT4 translocation induced by ischemic stimuli [5]. Although it has classically been believed that these two facilitated-diffusion GLUTs are the only isoforms responsible for glucose uptake in the heart, recent studies have indicated that SGLT1 is actually expressed in murine and human myocardia [9–11,28–32], which is confirmed by the present findings (Figs 2 and 3), and facilitates myocardial glucose uptake[10,28,32]. Moreover, Banerjee et al. demonstrated that the SGLT1 expression is increased under both conditions of ischemic or diabetic cardiomyopathy, possibly as an adaptive response to cardiac damage [10], while the same group and others reported that chronic excessive activation of cardiac SGLT1 has unfavorable effects [31,32], thus suggesting that the time course of SGLTs activation is critical for eliciting cardioprotective effects. Although the plasma membrane expression level of SGLT1 was not affected during a short period of IRI in the present study, our results indicate the possible role of SGLT1 activation (in addition to its translocation/internalization-mediated expression level), considering that phlorizin has a significant effect on the cardiac functional recovery and injury after ischemia-reperfusion, whereas no significant effects were noted on the baseline cardiac function. In fact, previous studies have demonstrated that the activation of SGLT1 in the myocardium induced by insulin stimulation exerts positive inotropic effects, indicating that SGLT1 actually has substantial functional effects in the presence of stimulatory factors [29,30]. In contrast to facilitated energy-independent GLUT transport, which may become inefficient under conditions of a low extracellular glucose concentration, SGLTs, as active transporters, work against the glucose concentration gradient by coupling glucose transport to the downhill Na^+^ electrochemical gradient via Na^+^/K^+^ATPase and thus are essential for cardiomyocyte survival in environments with a low glucose concentration, such as that associated with ischemia [11,12,37]. Therefore, SGLT1 activation in the heart plays a particularly important role under critical pathological conditions relative to baseline conditions.

We cannot rule out the potential impact of phlorizin on endothelial transport or function, rather than myocardial glucose metabolism, since SGLT1 is also expressed in endothelial cells [25,27]. On the other hand, the present study, together with previous studies by other groups demonstrated that SGLT1 is substantially expressed in the myocardium [10] and actually contributes to the pathogenesis of certain types of cardiac diseases, such as PRKAG2 cardiomyopathy [28] [32]. SGLT1 also exerts functional effects in the myocardium [29–31]. Therefore, the effects of phlorizin on the heart would be expected to be largely mediated through myocardial SGLT1 in the current observations, although endothelial SGLT1 may have contributed to the observed effects.

Anaerobic glycolysis appears to be particularly important in replenishing ATP stores in the heart following exposure to ischemia [1,5,34,36]. However, the mechanism underlying the reduction of the ATP content at the end of the pre-ischemic period in the phlorizin-perfused heart remains uncertain, although it was not associated with the reductions in cardiac function, glucose uptake, lactate output, or glycogen content at baseline. To the best of our knowledge, there are only a few reports indicating non-specific SGLTs-independent effects of phlorizin on mitochondrial energetics [38,39]. Phlorizin induces mitochondrial swelling [38], and also inhibits mitochondrial ATPase activity [39], leading to the decrease in the mitochondrial ATP level. These findings could explain one of the possible mechanisms of the reduction of the ATP content in the phlorizin-perfused hearts, especially under baseline normoxia settings in which myocardial ATP synthesis is relatively dependent on mitochondrial oxidative phosphorylation. In contrast, the significant reduction of the ATP content during the reperfusion period was associated with the reductions in glucose uptake and lactate output, which indicates the glycolytic flux in this particular model of the Langendorff heart perfusion setting [5,18,22,34]. The results obtained from these measurements showed lower glycolytic activity, supporting the hypothesis that there is impaired glucose delivery in the phlorizin-perfused hearts during IRI. Moreover, Cross et al. previously demonstrated that a decrease in lactate output coincided with the onset of ischemic contracture in the Langendorff model, although the hearts were subjected to low-flow ischemia, not global ischemia, in which lactate can accumulate [34]. These findings strongly support the idea that SGLT1 inhibition during IRI results in a reduction of myocardial glucose utilization, leading to an impairment in replenishing ATP stores in the heart.

Glucose was the only substrate used in the present *ex vivo* Langendorff perfusion model, which does not completely mimic physiological substrates *in vivo* [5]. This model involves an increase in the relative level of dependence on glucose in the heart, and the hearts are more susceptible to the effects of a low glucose concentration, although phlorizin perfusion during the pre-ischemic period (namely, under substantial amounts of glucose) does not affect the baseline cardiac function (Table 1). In this context, the Langendorff system is useful for evaluating the direct local effects of glucose transporters in the heart, as this system is not associated with changes in systemic substrate metabolism or neurohumoral factors activated under pathological conditions, which may influence the cardiac function and degree of myocardial injury.

A series of our recent studies of insulin signaling and the aldosterone cascade indicate that a transient increase in either glucose or sodium uptake into cardiomyocytes is actually cardioprotective under specific pathological conditions, such as IRI [3,4,15,20,40]. In this context, it is plausible that the activation of sodium-glucose cotransporter in the heart provides cardioprotective effects against IRI, at least during the acute phase, as observed in the present study. Moreover, we recently reported a transient decrease in the serum potassium level during ischemic attacks of acute coronary syndrome (ACS), the degree of which is positively correlated with plasma glucose level [41]. The larger potassium decrease observed in subjects with higher glucose levels during ischemic attacks suggests that SGLT1- Na^+^/K^+^ATPase coupling is at least partially involved in the pathophysiology of the acute phase of ACS as a compensatory protective mechanism.

There are several recently developed SGLT2 inhibitors used as novel-anti-diabetic agents [12,42]. In contrast to SGLT2, which is a low-affinity high-capacity glucose transporter, SGLT1 is a high-affinity low-capacity transporter [11,12,42,43]. Hence, it is likely that SGLT1 is predominant in the heart as a cardioprotective mechanism in low-glucose settings, such as that involving ischemia. Therefore, the current study focused on SGLT1, a substantial expression of which was confirmed in both the human and murine hearts directly on immunostaining as well as immunoblotting (Figs 2 and 3), whereas SGLT2 expression in the heart has yet to be determined definitively [9,11]. The effects of SGLT2 inhibition on the heart, either directly or indirectly via mutual interaction with SGLT1, remain unclear and warrant further examination [44].

To date, various actions of phlorizin have been reported, and some effects may be mediated through SGLT1-independent mechanisms (including the potential direct effects on mitochondria, as described above). The possible cardioprotective role of phlorizin as an anti-arrhythmic agent, as well as a free radical scavenger, cannot be excluded as reported by others [45], although a variety of important differences between the experimental animal species used (the study by Hirose et al. [43] used guinea pig hearts, in which SGLT1 expression has not yet been detected), the experimental conditions and the time course of phlorizin perfusion may have contributed to the differences in the observations. In addition, considering that the aglycone of phlorizin is actually a GLUTs inhibitor [46–48], and also that 2-DG, used for the glucose uptake measurement (Fig 6C), is thought to be a relatively poor substrate for SGLT1 (although 2-DG has been used for the glucose uptake assay of SGLTs in prior studies [49]), non-specific inhibitory effects of phlorizin on other glucose transporters might be at least partly involved in the findings observed in the current study. These are certain limitations of this study and future investigations will be directed at conducting a series of experiments using a SGLT1 knock out model in order to clarify more complete characterization of SGLT1 in the heart.

In summary, we confirmed that SGLT1 is substantially expressed in human hearts. We also demonstrated that phlorizin predisposes the heart to profound IRI, which was associated with a significant decrease in the cardiac tissue ATP content, possibly as a result of a reduction in the glucose uptake as well as the glycolytic flux in the heart (namely, myocardial glucose utilization) during ischemia-reperfusion. These results provide new insight into the important role of cardiac SGLTs, possibly SGLT1 in particular, in optimizing cardiac energy metabolism under specific pathological conditions, such as during the acute phase of IRI.
